# Conservative Management of a Ruptured Simple Hepatic Cyst: A Case Report

**DOI:** 10.7759/cureus.97594

**Published:** 2025-11-23

**Authors:** Abhilash Mesh, Chloe Thomas, Gayan Nanayakkara, Ahmed Aly, Mohammed Elmorsy

**Affiliations:** 1 Medicine, Withybush Hospital, Haverfordwest, GBR; 2 Surgery, Withybush Hospital, Haverfordwest, GBR; 3 General Surgery, Withybush Hospital, Haverfordwest, GBR

**Keywords:** conservative approach, cyst deroofing, hepatobiliary, liver cyst, perihepatic fluid collection, radiology, right upper quadrant abdominal pain, spontaneous hepatic cyst rupture, surgery

## Abstract

Simple hepatic cysts are common benign liver lesions that are typically asymptomatic and discovered incidentally. They are generally managed conservatively unless symptoms or complications develop. Spontaneous rupture of a hepatic cyst is exceptionally rare and often necessitates surgical or interventional management. We report the case of a 79-year-old woman with a large known hepatic cyst who presented with acute right upper quadrant pain, nausea, and vomiting. Imaging revealed rupture of the cyst with associated perihepatic free fluid, but no evidence of haemorrhage or infection. Given her haemodynamic stability and absence of sepsis, a conservative approach was adopted. She was managed with close observation, supportive care, and analgesia, without the need for surgical or percutaneous intervention. The patient remained clinically stable, her symptoms resolved gradually, and she was discharged home in good condition. This case demonstrates that conservative management can be a safe and effective option in carefully selected patients with ruptured simple hepatic cysts, particularly those who are haemodynamically stable and without evidence of infection or bleeding. Early imaging, multidisciplinary input, and vigilant clinical monitoring are key to successful non-operative outcomes.

## Introduction

Simple hepatic cysts are benign, epithelial-lined, fluid-filled lesions that arise within the liver parenchyma and represent the most common form of non-parasitic cystic liver disease [1]. They are usually congenital in origin, believed to result from biliary microhamartomas or aberrant bile duct development [2]. The reported prevalence of simple hepatic cysts ranges from 2.5% to 18%, with a higher incidence in women and a progressive increase with age [3,4].

Most hepatic cysts are asymptomatic and discovered incidentally during abdominal imaging performed for unrelated reasons [1,5]. When large, typically greater than 5-10 cm in diameter, they may cause non-specific symptoms such as right upper quadrant discomfort, early satiety, nausea, or abdominal distension secondary to compression of adjacent organs [5]. Rarely, large cysts can lead to complications including intracystic haemorrhage, infection, torsion, rupture, or biliary obstruction [5-7].

Among these, spontaneous rupture of a simple hepatic cyst is exceptionally uncommon, with only isolated cases described in the English-language literature [6,7]. The underlying mechanism remains unclear but is thought to involve a combination of progressive cyst enlargement, thinning of the cyst wall, ischaemic degeneration, or minor trauma [6,7].

Given the rarity of rupture, its clinical presentation can be challenging to differentiate from other acute abdominal conditions such as cholecystitis, perforated peptic ulcer, or pancreatitis. Imaging plays a critical diagnostic role; while ultrasonography provides initial assessment, contrast-enhanced computed tomography (CT) remains the modality of choice, typically demonstrating cyst wall discontinuity, interval size reduction, and perihepatic fluid consistent with rupture [5-7].

Management strategies depend on the patient’s clinical status. Conservative management may be appropriate in haemodynamically stable patients without infection or haemorrhage, while surgical deroofing or hepatic resection is reserved for recurrent, symptomatic, or complicated cases [5].

We present the case of an elderly woman who developed acute abdominal pain secondary to spontaneous rupture of a large simple hepatic cyst. She was managed conservatively with excellent short-term recovery before undergoing elective laparoscopic deroofing as definitive treatment. This case illustrates the importance of early recognition, the potential role of conservative management in selected patients, and the value of multidisciplinary decision-making in achieving optimal outcomes [1-9].

## Case presentation

A previously well 79-year-old woman presented to her general practitioner with a one-week history of right-sided flank discomfort and a sensation of abdominal tightness, most noticeable when dressing. The discomfort had progressively worsened over the preceding days. She had self-medicated with oral tramadol, which provided partial symptom relief. Associated symptoms included intermittent nausea, occasional retching, and loss of appetite, although bowel habits remained normal. She also reported generalised fatigue and intermittent dizziness but denied fever, jaundice, urinary symptoms, or alteration in bowel habit.

Her past medical history included hypertension and osteoarthritis. Surgical history comprised an umbilical hernia repair approximately 40 years earlier, bilateral total knee replacements, a total hip replacement with subsequent revision, and an osteotomy. She was not taking anticoagulants and had no history of chronic liver disease or alcohol misuse. She had not previously been diagnosed with any hepatic pathology, and there was no prior imaging suggestive of a liver cyst.

She was referred to the local Same-Day Emergency Care (SDEC) unit for further evaluation. On examination, she appeared comfortable and was haemodynamically stable. Abdominal examination revealed significant hepatomegaly with tenderness on palpation across the right upper quadrant and extending centrally, but without rigidity, guarding, or rebound tenderness. The remainder of the systemic examination was unremarkable.

Initial laboratory investigations (Table 1) demonstrated a mild inflammatory response without evidence of infection, hepatic dysfunction, or coagulopathy. Haemoglobin and white cell counts were within normal limits, excluding acute haemorrhage or sepsis, while a markedly elevated C-reactive protein (CRP) (106 mg/L) indicated localised inflammation consistent with cyst rupture. Liver enzymes and bilirubin remained normal, confirming preserved hepatic function and the absence of biliary obstruction. Renal function was largely maintained, though the estimated glomerular filtration rate (eGFR) (52 mL/min/1.73 m²) reflected mild, likely age-related impairment. Coagulation parameters were normal, but fibrinogen was elevated (6.8 g/L), supporting an acute-phase response. Overall, the results were consistent with a stable inflammatory process amenable to conservative management. She was discharged home with analgesia and scheduled to return the following day for a formal abdominal ultrasound.

**Table 1:** Intial blood investigations eGFR: estimated glomerular filtration rate, APTT: activated partial thromboplastin time; INR: international normalised ratio

Parameter	Result	Reference Range	Units
Haemoglobin	131	115–165	g/L
White cell count	9.1	4.0–11.0	×10⁹/L
Platelet count	402	150–400	×10⁹/L
C-reactive protein	106	<5	mg/L
Aspartate transaminase	31	<32	U/L
Alanine transaminase	17	<33	U/L
Alkaline phosphatase	137	30–130	U/L
Bilirubin	16	<21	µmol/L
Albumin	48	35–50	g/L
Total protein	80	60–80	g/L
Calcium	2.44	2.20–2.60	mmol/L
Adjusted calcium	2.39	2.20–2.60	mmol/L
Urea	6.3	2.5–7.8	mmol/L
Creatinine	91	46–92	µmol/L
eGFR	52	>60	mL/min/1.73 m²
Sodium	137	133–146	mmol/L
Potassium	3.6	3.5–5.3	mmol/L
Prothrombin time	12.1	9.0–13.0	s
APTT	28.1	27.0–36.4	s
INR	1	0.8–1.2	ratio
Fibrinogen	6.8	2.0–4.0	g/L

The following day, the patient re-presented to the emergency department with sudden-onset, severe right upper quadrant abdominal pain. The pain was sharp, constant, and rated 10/10 in intensity. It had developed abruptly several hours earlier and was associated with bilious vomiting, subjective fever, and chills. She denied trauma, recent abdominal procedures, or anticoagulant use.

On arrival, she was alert and oriented with a Glasgow Coma Scale (GCS) score of 15/15. Vital signs were as follows: temperature 36.9 °C, heart rate 101 beats per minute, respiratory rate 16 breaths per minute, blood pressure within normal limits, and oxygen saturation 96% on room air. She was afebrile and haemodynamically stable.

Abdominal examination demonstrated mild tenderness localised to the right upper quadrant without guarding, rigidity, or rebound tenderness. There was no palpable hepatomegaly or abdominal mass, and the liver edge was no longer appreciable, suggesting interval decompression of the cyst. There were no stigmata of chronic liver disease. Cardiovascular and respiratory examinations were normal.

Laboratory results on Day 3, obtained at the time of clinical deterioration (Table 2), demonstrated an acute inflammatory response. The white cell count was elevated at 13.5 × 10⁹/L, with marked neutrophilia (12.5 × 10⁹/L) and lymphopenia (0.5 × 10⁹/L), consistent with physiological stress. Haemoglobin (118 g/L) and platelets (384 × 10⁹/L) remained within normal limits, excluding bleeding or marrow suppression. Liver function tests were normal, confirming preserved hepatic function despite cyst rupture. CRP was raised at 70 mg/L, reflecting localised inflammation without systemic sepsis. Renal function was normal (creatinine 61 µmol/L, eGFR 82 mL/min/1.73 m²), with mild hypokalaemia (3.1 mmol/L). Coagulation parameters were normal, while fibrinogen was elevated (5.4 g/L), consistent with an acute-phase response. Overall, the results supported a localised inflammatory process secondary to cyst rupture, without hepatic dysfunction or sepsis.

**Table 2:** Blood investigations on Day 3, at the time of the patient’s acute clinical deterioration eGFR: estimated glomerular filtration rate, APTT: activated partial thromboplastin time

Parameter	Result	Reference Range	Units
White blood cell count	13.5	4.0 – 11.0	×10⁹/L
Neutrophil count	12.5	1.7 – 7.5	×10⁹/L
Lymphocyte count	0.5	1.0 – 4.5	×10⁹/L
Monocyte count	0.5	0.2 – 0.8	×10⁹/L
Eosinophil count	0	0.0 – 0.4	×10⁹/L
Basophil count	0	0.0 – 0.1	×10⁹/L
Haemoglobin	118	115 – 165	g/L
Red blood cell count	3.76	3.80 – 5.50	×10¹²/L
Haematocrit	0.36	0.37 – 0.47	L/L
Mean cell volume	97	80 – 100	fL
Mean cell haemoglobin	31	27 – 33	pg
Red cell distribution width	12.7	11.0 – 14.8	%
Platelet count	384	150 – 400	×10⁹/L
Prothrombin time	13.2	9.0 – 13.0	sec
APTT	22.6	27.0 – 36.4	sec
Clauss fibrinogen level	5.4	2.0 – 4.0	g/L
Bilirubin	10	<21	µmol/L
Total protein	62	60 – 80	g/L
Albumin	40	35 – 50	g/L
Globulin	22	20 – 35	g/L
Alkaline phosphatase	97	30 – 130	U/L
Alanine transaminase	8	<33	U/L
C-reactive protein	70	<5	mg/L
Urea	7.4	2.5 – 7.8	mmol/L
Creatinine	61	46 – 92	µmol/L
eGFR	82	≥60	mL/min/1.73 m²
Sodium	137	133 – 146	mmol/L
Potassium	3.1	3.5 – 5.3	mmol/L

**Figure 1:**
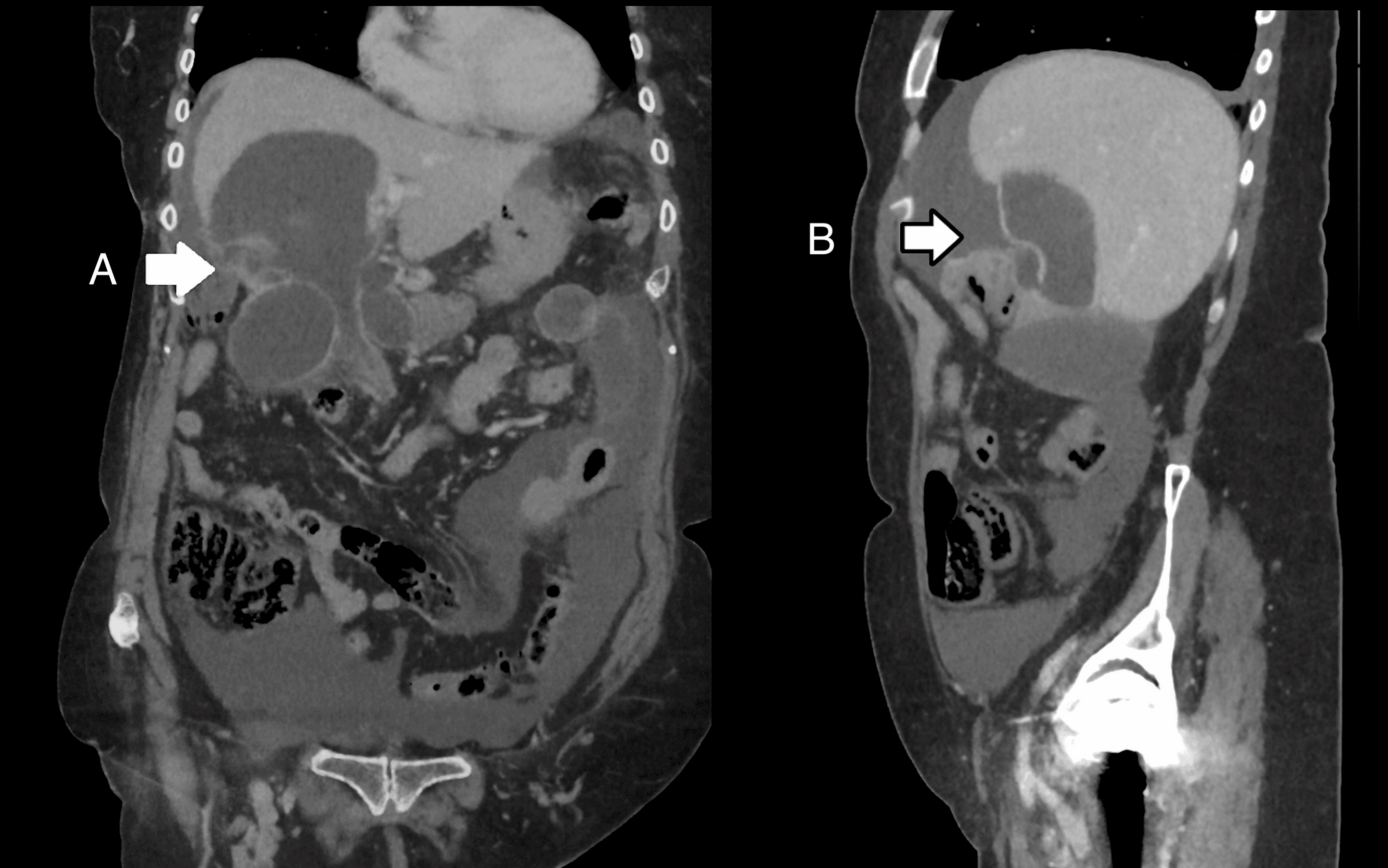
CT Imaging (A) Coronal CT image showing a ruptured hepatic cyst (white arrow) with surrounding low-attenuation fluid tracking along the peritoneal cavity, consistent with cyst leakage. (B) Sagittal CT image further demonstrating the disrupted cyst wall (white arrow) and associated perihepatic fluid collection, supporting the diagnosis of cyst rupture.

CT scan of the abdomen and pelvis demonstrated a marked reduction in the size of the right hepatic cyst to 8.1 × 6.8 × 10 cm, with partial collapse of its lateral wall and a large, low-attenuation perihepatic fluid collection. These findings were consistent with spontaneous rupture of the cyst and extravasation of intracystic fluid into the peritoneal cavity. The residual cyst wall appeared thin and irregular, without mural enhancement, haemorrhage, bile leak, or contrast extravasation. There was no radiological evidence of malignancy, intra-abdominal sepsis, or biliary obstruction. The coronal and sagittal views demonstrated the collapsed cyst cavity and associated perihepatic fluid collection (Figure 1).

The case was discussed with the regional hepatobiliary surgical team, and given the patient’s advanced age, multiple comorbidities, and sustained haemodynamic stability, a conservative, non-operative approach was deemed the most appropriate course of action. There was no radiological or biochemical evidence of infection, haemorrhage, or biliary communication, and the patient exhibited no clinical signs of sepsis or peritonitis. In light of these findings, surgical or percutaneous intervention was deferred in favour of close observation and supportive therapy.

The patient was admitted under the surgical team for inpatient monitoring. Conservative management focused on maintaining haemodynamic stability, controlling pain, and preventing complications. She received intravenous fluids for hydration, regular analgesia for symptom control, and venous thromboembolism prophylaxis. Antibiotics were intentionally withheld, as there was no evidence of bacterial infection or cyst superinfection. Serial blood investigations demonstrated stable haemoglobin and liver function, with a gradual decline in inflammatory markers, supporting the absence of ongoing bleeding or infection.

During her hospital stay, the patient experienced a brief, self-limiting episode of diarrhoea, but stool cultures and *Clostridioides difficile* toxin assays were negative. Her abdominal pain gradually subsided, her appetite improved, and she remained afebrile and haemodynamically stable throughout her admission. Regular clinical assessments confirmed the resolution of tenderness, and there was no progression towards peritonitis or sepsis.

**Figure 2:**
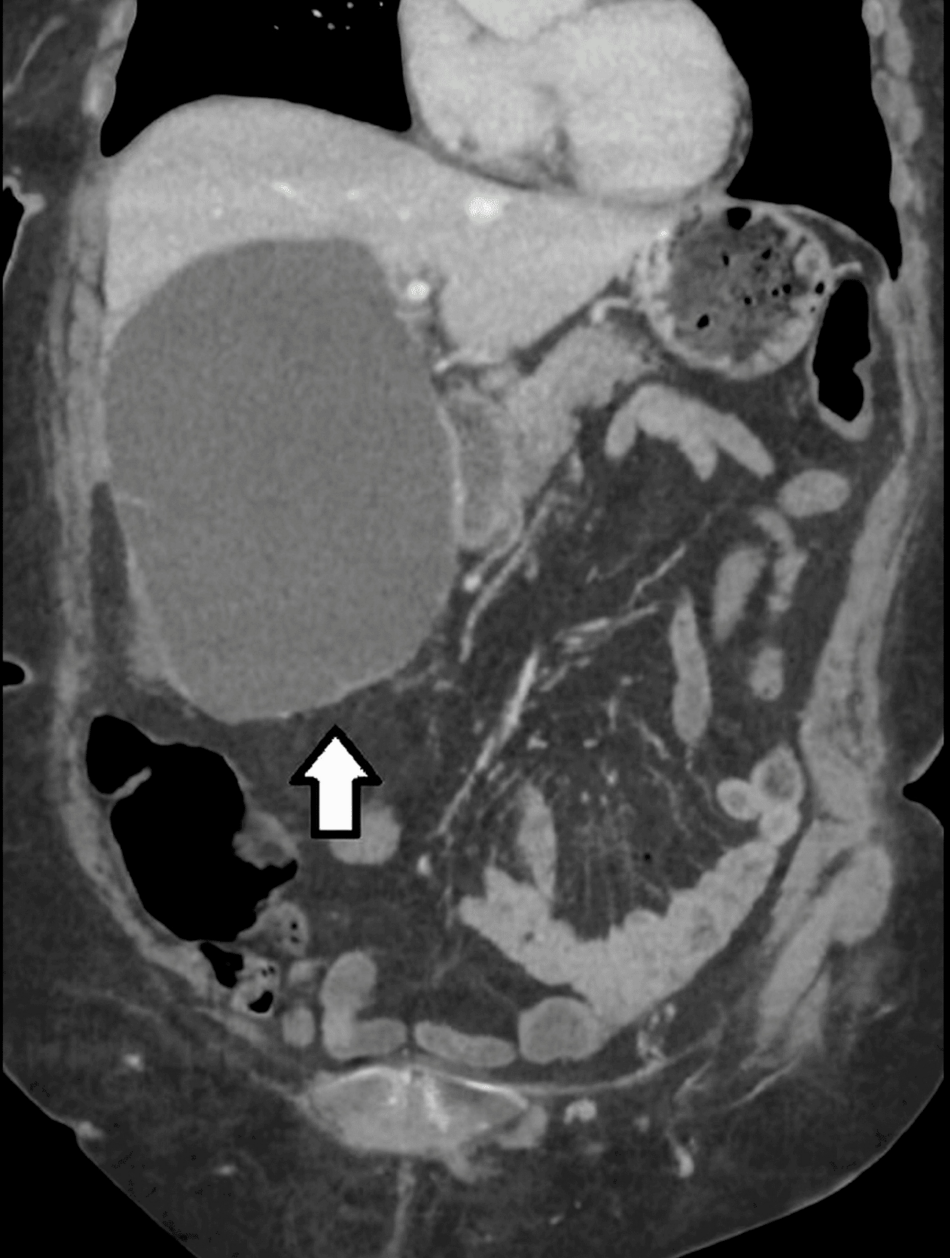
Coronal CT image The white arrow indicates reaccumulation of homogeneous, low-attenuation fluid within the cavity of the ruptured hepatic cyst, consistent with sterile fluid collection.

A repeat contrast-enhanced CT scan performed four days later demonstrated interval enlargement of the perihepatic fluid collection, now measuring 12.5 × 9 × 15.5 cm. The collection remained homogeneous and of low attenuation, without gas, rim enhancement, or internal septations-features consistent with sterile cyst fluid rather than abscess or haematoma. No evidence of biliary obstruction or intrahepatic ductal dilatation was observed (Figure 2).

By the end of the first week, the patient was asymptomatic, with a soft, non-tender abdomen and normal vital signs. Given her marked clinical improvement, repeat imaging was deferred, as there was no indication of further complications or fluid reaccumulation. She was discharged following a short inpatient stay with clear safety-netting advice, including instructions to seek urgent medical review if symptoms recurred. Outpatient hepatobiliary follow-up was arranged for ongoing assessment and discussion of elective management options. 

Subsequently, she was referred to the regional hepatobiliary unit, where she underwent an elective cyst deroofing procedure for definitive management. She underwent laparoscopic deroofing of a large symptomatic hepatic cyst arising from segment IV of the liver, with its lateral wall abutting the medial aspect of the gallbladder. Pneumoperitoneum was established using an open (Hasson) technique due to previous midline surgery, and three ports were inserted under direct vision. Extensive adhesiolysis was required to expose the cyst, which was then punctured and aspirated, evacuating its clear serous contents. Deroofing of the cyst wall was performed using a harmonic scalpel, and the resected portion of the cyst wall was retrieved in an endoscopic retrieval bag. Haemostasis was achieved with minimal blood loss, and an omental patch was placed within the residual cavity and secured with clips to prevent recurrence. All port sites were closed in layers.

The patient tolerated the procedure well, with no intraoperative complications. Postoperatively, she made an uneventful recovery; oral intake and mobilisation were commenced on the same day. Liver function tests remained within normal limits, and she was discharged 48 hours after surgery in good condition. At follow-up, the patient remained asymptomatic, with no evidence of recurrence or postoperative complications on clinical review and follow-up imaging.

## Discussion

Simple hepatic cysts are common benign liver lesions with a reported prevalence ranging from 2.5% to 18% in the general population, occurring more frequently in women and often identified incidentally during imaging for unrelated conditions [1,5]. Although most cysts remain asymptomatic, complications such as infection, haemorrhage, biliary obstruction, or rupture may occur [5-9]. Spontaneous rupture is exceptionally rare, with only a few cases reported, predominantly in elderly women [6,7]. The mechanism of rupture remains uncertain but is thought to involve rapid cyst enlargement, elevated intracystic pressure, and wall weakening due to haemorrhage, infection, or ischaemia, occasionally exacerbated by external trauma [5-7]. In the present case, the absence of haemorrhage or infection suggests that progressive distension and wall thinning were the likely causes.

Clinical presentation is often non-specific and can mimic other acute abdominal conditions such as acute cholecystitis, perforated peptic ulcer, or pancreatitis [5-7]. Imaging plays a critical diagnostic role, with ultrasonography serving as a useful first-line tool, though it may be limited by cyst size or overlying bowel gas [5]. Contrast-enhanced CT is considered the diagnostic gold standard, typically revealing cyst wall discontinuity, reduction in cyst size, and perihepatic low-attenuation fluid, while hyperdense peritoneal fluid or intracystic debris may indicate haemorrhage [6,7]. There are no standardised management guidelines due to the rarity of spontaneous rupture [5-9].

Treatment decisions depend on haemodynamic stability, cyst characteristics, and associated complications. Surgical options such as laparoscopic or open deroofing, cyst excision, or partial hepatic resection offer definitive treatment but carry perioperative risks, especially in elderly or comorbid patients [5]. Percutaneous aspiration with or without sclerotherapy provides temporary relief but is associated with a high recurrence rate and infection risk [5,6]. Conservative management is appropriate for stable patients without haemorrhage or infection, focusing on supportive therapy and close observation [5,7]. In this case, conservative management led to symptomatic and biochemical improvement, followed by elective laparoscopic deroofing for definitive resolution. This experience highlights that a non-operative approach can serve as a safe and effective bridge to elective surgery in stable patients, minimising emergency intervention risks and allowing multidisciplinary planning for optimal outcomes.

Lessons learnt

This case underscores several important clinical lessons in the recognition and management of ruptured simple hepatic cysts. Rupture of simple hepatic cysts, though rare, should be considered in elderly patients presenting with acute upper abdominal pain and a history of hepatic cysts. The presentation can mimic more common causes such as cholecystitis, peptic ulcer perforation, or pancreatitis, and a high index of suspicion is therefore required [5-7]. Contrast-enhanced CT imaging is the diagnostic modality of choice, demonstrating characteristic findings such as wall discontinuity, interval cyst size reduction, and associated peritoneal fluid. Ultrasonography may identify cystic structures but is often limited in differentiating rupture or detecting subtle wall defects [5-7].

Conservative management can be a safe and effective initial approach in haemodynamically stable patients without evidence of haemorrhage, sepsis, or biliary communication. Supportive care, including intravenous fluids, analgesia, and observation, may result in spontaneous resolution and symptom improvement [5], as demonstrated in this case. Elective laparoscopic deroofing remains the definitive treatment for large, recurrent, or symptomatic cysts. Surgical intervention under controlled, elective circumstances minimises operative risk, particularly in elderly or comorbid patients, and provides durable resolution with low recurrence rates [5-8]. Multidisciplinary decision-making is essential, involving hepatobiliary surgeons, radiologists, and physicians to tailor management based on clinical stability, cyst characteristics, and patient comorbidities [5].

Conservative management proved successful in this case, illustrating that non-operative treatment can be an appropriate option in carefully selected patients, particularly those with extensive prior abdominal surgery or higher operative risk. The favourable outcome supports a stepwise approach when the patient is clinically stable and the cyst shows no signs of infection, torsion, or malignant potential. Nonetheless, mesenteric cysts carry a recognised risk of recurrence, reaccumulation, or repeat rupture, which may later necessitate surgical intervention. This possibility should be clearly communicated to patients, as ongoing clinical follow-up is essential to detect early signs of deterioration or recurrence. Including such considerations ensures a more balanced understanding of conservative care, acknowledging both its benefits and limitations.

By reporting this case, we highlight the importance of individualised decision-making and reinforce the need to weigh surgical risks against the natural behaviour of the cyst, particularly in patients with complex surgical histories.

## Conclusions

Conservative management can be a safe and effective initial approach for haemodynamically stable patients with ruptured simple hepatic cysts in the absence of haemorrhage or infection. It allows clinical stabilisation and facilitates planned elective surgery under optimal conditions. An individualised, multidisciplinary strategy remains essential to achieving favourable outcomes and minimising unnecessary surgical morbidity in this rare clinical scenario.
